# Relationship between academic procrastination, self-esteem, and moral intelligence among medical sciences students: a cross-sectional study

**DOI:** 10.1186/s40359-024-01731-8

**Published:** 2024-04-23

**Authors:** Saeed Ghasempour, Aliasghar Babaei, Soheil Nouri, Mohammad Hasan Basirinezhad, Ali Abbasi

**Affiliations:** 1 Student Research Committee, School of Nursing and Midwifery, Shahroud University of Medical Sciences, Shahroud, Iran; 2 Department of Epidemiology and Biostatistics, School of Public Health, Shahid Sadoughi University of Medical Sciences, Yazd, Iran; 3 Department of Nursing, School of Nursing and Midwifery, Shahroud University of Medical Sciences, Shahroud, Iran

**Keywords:** Academic procrastination, Self-esteem, Moral intelligence, Students

## Abstract

**Background:**

Academic procrastination is a widespread phenomenon among students. Therefore, evaluating the related factors has always been among the major concerns of educational system researchers. The present study aimed to determine the relationship of academic procrastination with self-esteem and moral intelligence in Shahroud University of Medical Sciences students.

**Methods:**

This cross-sectional descriptive-analytical study was conducted on 205 medical sciences students. Participants were selected based on inclusion and exclusion criteria using the convenience sampling technique. The data collection tools included a demographic information form, Solomon and Rothblum’s Procrastination Assessment Scale-Students, Rosenberg Self-Esteem Scale, and Lennick and Kiel’s Moral Intelligence Questionnaire, all of which were completed online. The data were analyzed using descriptive statistics and inferential tests (multivariate linear regression with backward method) in SPSS software.

**Results:**

96.1% of participating students experienced moderate to severe levels of academic procrastination. Based on the results of the backward multivariate linear regression model, the variables in the model explained 27.7% of the variance of academic procrastination. Additionally, self-esteem (*P <* 0.001, *β=*-0.942), grade point average (*P <* 0.001, *β=*-2.383), and interest in the study field (*P =* 0.006, *β=*-1.139) were reported as factors related to students’ academic procrastination.

**Conclusion:**

According to the findings of this study, the majority of students suffer from high levels of academic procrastination. Furthermore, this problem was associated with low levels of self-esteem, grade point average, and interest in their field of study.

## Introduction

Investigating factors associated with students’ academic performance has always been the focus of researchers in the education system [1–3]. One of these factors is academic procrastination, which is a common phenomenon among students [4–6]. This particular type of postponement refers to learners’ dominant and constant tendency to postpone academic tasks such that it affects their anticipated performance [6]. In general, two types of procrastination are observed in students’ homework. One type is purposeful, planned, and thoughtful postponement. For example, when students have to complete many assignments simultaneously, they prioritize some important assignments. Another type is irrational, self-defeating, and harmful postponement, which is known as academic procrastination [7]. Rothblum et al. (1986) propose two criteria in the definition and diagnoses of this problem: (a) tendency to always or almost always discard academic assignments and (b) always or almost always experiencing anxiety caused by such behavior. They emphasize that academic procrastination should include frequent postponement and considerable anxiety [8]. Research has shown that at least 70% of students are somehow involved in academic procrastination, and 50% always procrastinate in doing homework and learning course materials [9, 10]. These figures redouble the need to evaluate academic procrastination and its related factors in this group.

Academic procrastination is a complex concept that depends on some factors. These factors are both affected by academic procrastination and can also decelerate its process. Therefore, it is essential to identify the underlying factors affecting students’ academic procrastination [11]. In addition, academic procrastination and related factors have not yet been well investigated and require more studies, especially among medical students [12].

Moreover, academic procrastination is associated with high levels of anxiety, depression, and feeling guilty in students and affects their self-esteem [13–15]. Self-esteem is considered among the factors affecting students’ academic procrastination in various studies [16–18]. Self-esteem refers to our perception of ourselves, how we evaluate ourselves, and our self-evaluation of ourselves as individuals [19–21]. Coopersmith (1990) considers self-esteem as people’s evaluation of their worth and usually maintains, indicating an attitude of approval or disapproval. In other words, self-esteem is a personal judgment of one’s worth, which refers to a person’s feelings about their worth in various areas of life [22]. As one of the major factors that moderate psychosocial pressure, this concept forms based on family relationships, academic success, body image, social interaction, and sense of self-worth. In this respect, the importance of these contexts changes depending on individual differences and one’s growth [23].

Moral intelligence is another factor affecting students’ academic procrastination [24]. Moral intelligence is the capacity and ability to understand good issues from bad issues [25]. Indeed, this intelligence enhances appropriate behavior and can provide stability in social life over time through qualities (e.g., honesty, responsibility, forgiveness, and sympathy) and reduce misbehaviors. Moral intelligence reflects the fact that a person is not born moral or immoral but must learn good performance, conscientiousness, and responsibility [26]. According to Lennick and Kiel (2007), moral intelligence includes four principles: honesty, responsibility, forgiveness, and sympathy. The honesty principle refers to harmonization between people’s beliefs and actions. The responsibility principle is the acceptance of actions and their consequences, as well as mistakes and failures. The forgiveness principle includes awareness of faults and mistakes and forgiving oneself and others. Finally, the sympathy principle means paying attention to others [27].

As previously mentioned, academic procrastination is a prevalent phenomenon among students. Determining the factors associated with it has captured the attention of many researchers in the education system. However, there are limited studies on the relationship between psychological variables, such as self-esteem and moral intelligence, with academic procrastination. It seems that understanding the relationship between them will lead to providing appropriate solutions and approaches to reduce this problem and improve students’ academic performance. Therefore, since no study has been conducted to determine the relationship between these variables, this study aimed to investigate the relationship between academic procrastination, self-esteem, and moral intelligence among medical sciences students.

## Materials and methods

### Study design and participants

This descriptive-analytical study was conducted on 205 Shahroud University of Medical Sciences students from April to September 2023. Participants were included in the study based on inclusion and exclusion criteria through the convenience sampling technique. This technique was chosen for its ease of implementation, high response rate to questionnaires, and frequent use in similar studies [28].

The inclusion criteria were studying at bachelor and professional doctorate levels (no history of studying in other universities) and having theoretical and practical courses. Besides, exclusion criteria were the history of suffering from serious mental illnesses (SMI) (such as Major Depression Disorder (MDD), Schizophrenia, Bipolar Disorder (BD), Obsessive-Compulsive Disorder (OCD), Post-Traumatic Stress (PTSD), and other related disorders), using neuropsychological drugs (e.g., antidepressants, antipsychotics, anti-anxiety, and mood stabilizers), and the recent occurrence of unfortunate events or stressful events in the past six months, which was self-reported by the student.

The sample size was estimated to be 205 students based on the study by Uma et al. (2020) [29]. This estimation took into account a power of 90% at a confidence level of 95%, as well as a 15% attrition rate.

α = 0.05 β = 0.10 *r* = 0.24

$${\rm{n = }}{\left[ {\frac{{{Z_{1 - {\raise0.7ex\hbox{$\alpha $} \!\mathord{\left/{\vphantom {\alpha 2}}\right.\kern-\nulldelimiterspace}\!\lower0.7ex\hbox{$2$}}}} + {Z_{1 - \beta }}}}{{\frac{{\rm{1}}}{{\rm{2}}}{\rm{ log}}\frac{{{\rm{1 + r}}}}{{{\rm{1 - r}}}}}}} \right]^{\rm{2}}}{\rm{ + 3 = 178}}$$

### Measurements

The data collection tool in this study consisted of four sections designed using the DigiSurvey system, a web-based questionnaire tool (https://www.digisurvey.net/). The study objectives, along with the created link, were shared with students in their respective groups and channels on Telegram and WhatsApp social networks for them to complete in their free time.

#### Section 1. Demographic information form

Information related to gender, age, marital status, field of study, academic semester, previous semester grade point average (GPA), interest levels in the field of study, study hours, parent’s education, and student’s place of residence were asked in this form.

#### Section 2. Solomon and Rothblum’s Procrastination Assessment Scale-Students (PASS)

Students’ academic procrastination was measured using the PASS. It consists of 27 items that examine three components, namely preparation for exams (items 1–6), preparation for assignments (items 9–17), and preparation for end-semester papers (items 20–25). In this study, two sets of questions were presented after each component: The first three questions (items 7, 18, and 26) measure the student’s feelings and emotions about procrastination. The second three questions (items 8, 19, and 27) assess their tendency to change the procrastination habit. The scoring criteria for the items are based on a 5-point Likert scale, including “never” (1), “rarely” (2), “sometimes” (3), “often” (4), and “always” (5). Items 4, 6, 11, 15, 16, 21, 23, and 25 are scored reversely. The scores of this scale range between 27 and 135, with scores of 27–62 indicating mild procrastination, 63–98 moderate procrastination, and 99–135 severe procrastination [18]. Solomon and Rothblum (1984) reported the reliability and internal consistency of 0.79 and 0.84, respectively, for this scale using Cronbach’s alpha method. The validity of the construct was assessed using factor analysis, and the results confirmed the acceptable validity of this scale. Besides, this scale was significantly correlated with the Beck Depression Inventory (BDI) (*P <* 0.0005, *r =* 0.44), Ellis’s Assessment Test for Irrational Belief (ATIB) (*P <* 0.0005, *r =* 0.30), and Rosenberg’s Self-Esteem Scale (RSES) (*P <* 0.0005, *r=*-0.23) [30]. Roshanzadeh et al. (2021) studied the psychometrics of the Persian version of this scale and calculated a Cronbach’s alpha coefficient of 0.87, suggesting its acceptable reliability. The validity of this scale was also investigated by confirmatory factor analysis (CFA). The results confirmed an acceptable fit for the structure of this scale, and all the goodness of fit (GoF) indices properly confirmed the model [30]. In the present study, the reliability of the Persian version of this scale was obtained at 0.85 by Cronbach’s alpha method.

#### Section 3. Rosenberg Self-Esteem Scale (RSES)

Students’ self-esteem was evaluated by RSES, a ten-item scale developed by Rosenberg (1965). This scale measures one’s positive and negative feelings about oneself. Although RSES is a single-factor scale, two positive and negative factors have been emphasized in this scale in the past years [31]. This scale is scored using several proposed methods, some of which score it as a four-option spectrum (completely agree to completely disagree) and others as two options (I agree and I disagree). The second form of this scale has been prepared in the Persian version in Iran, which is scored as “I agree” and “I disagree”. In this scale, + 1 and − 1 scores are respectively assigned to each “I agree” answer and each “I disagree” answer in questions 1–5. Questions 6–10 are scored in reverse, i.e., + 1 and − 1 scores are respectively assigned to each “I agree” answer and each “I disagree” answer in questions 6–10. Scores + 10, >0, <0, and − 10 indicate very high, high, low, and very low self-esteem levels, respectively [32, 33]. Rosenberg (1956) proposed this scale as a simple and short tool with appropriate reliability (internal consistency and re-test) and validity (convergent and divergent) [34]. Researchers claim this scale is a two-dimensional construct of positive and negative self-images when using the CFA. Besides, five items with positive words on one factor were named “positive self-esteem” (PSE), and five items with negative words on another factor were termed “negative self-esteem” (NSE) [35, 36]. Previous studies have reported a poor fit for the single-factor model of this scale using the CFA, as well as a better fit with positive and negative self-images for the two-factor model [37]. The Persian version of this scale was presented in a psychometric study [38]. The items’ internal similarity coefficients were obtained at 0.84, 0.87, and 0.80 for the whole sample, male students and female students, respectively. In addition, the correlation coefficients between each item of this scale and the total score of the items ranged from 0.56 to 0.72, and all were statistically significant at the 0.01 level. The CFA using principal axis factorization (Promax rotation) in the above scale resulted in two factors of personal competence and capability (items 4, 5, 7, 8, 9, and 10) and self-satisfaction (items 1, 2, 3, and 6), which explained 53.83% of the variance of the scale. Furthermore, a significant negative relationship was observed between the RSES and the Death Obsession Scale (DOS) in the whole sample (-0.34), in male students (-0.44), and in female students (-0.27), indicating the divergent validity of this scale [38]. In the present study, the reliability of the Persian version of this scale was calculated by Cronbach’s alpha method (0.82).

#### Section 4. Lennick and Kiel’s Moral Intelligence Questionnaire (MIQ)

The students’ moral intelligence was evaluated using Lennick and Kiel’s MIQ, which consists of 20 items examining four subscales, namely honesty (items 1–6), responsibility (items 7–12), forgiveness (items 13–16), and sympathy (items 17–20). Items are scored based on a 5-point Likert scale (never = 1, rarely = 2, sometimes = 3, oftentimes = 4, and always = 5). The scores of this questionnaire range between 20 and 100, with scores 20–33, 34–66, and >67 indicating low, average, and high moral intelligence levels, respectively [27]. Shahbaziyan et al. (2019) reported a Cronbach’s alpha coefficient of 0.91 for the reliability of the Persian version of this questionnaire, indicating a favorable level. The validity of this questionnaire was assessed by the CFA, where the good fit of the model revealed its favorable validity [24]. In the present study, the reliability of the Persian version of this questionnaire was obtained at 0.94 using Cronbach’s alpha method.

### Ethical considerations

The necessary permits were obtained from the Vice-Chancellor for Research and Technology and the Research Ethics Council of Shahroud University of Medical Sciences (Code of Ethics: IR.SHMU.REC.1402.043). Additionally, necessary arrangements were made with the officials of all four faculties, namely nursing and midwifery, medicine, paramedicine, health, and the heads of each field of study. Afterwards, the study objectives and relevant link were posted on social networks such as Telegram and WhatsApp within the students’ study groups and channels. They were asked to complete it in their free time.

### Statistical analysis

The data were analyzed using descriptive statistics (frequency, percentage, mean, and standard deviation) and inferential tests (multivariate linear regression with backward method) in SPSS software, with a significance level of 0.05.

## Results

In this study, most participants were female (64.4%) and single (95.1%). The mean and standard deviation of the participants’ age and GPA were 22.39 ± 2.21 and 16.65 ± 1.40, respectively. Other demographic characteristics of participating students are listed in Table 1. The participants’ average academic procrastination, self-esteem, and moral intelligence scores were 81.13 ± 89.06, 5.4 ± 03.84, and 78.87 ± 12.31, respectively. Table 2 presents the averages of these scores based on their subscales.

**Table 1:** The demographic characteristics of participating students

Variables		N (%)
Gender	Male	73 (35.6)
	Female	132 (64.4)
Marital status	Single	195 (95.1)
	Married	10 (4.9)
Field of study	Medical	45 (22.0)
	Nursing	52 (25.4)
	Midwifery	31 (15.1)
	Anesthesiology	13 (6.3)
	Operating Room	9 (4.4)
	Laboratory Science	15 (7.3)
	HIT	7 (3.4)
	Radiology	16 (7.8)
	Public Health	7 (3.4)
	Occupational Health Engineering	3 (1.5)
	Environmental Health Engineering	7 (3.4)
Mother’s education	Below diploma	58 (28.3)
	Diploma	72 (35.1)
	Associate	6 (2.9)
	BSc	52 (25.4)
	MSc	16 (7.8)
	Doctorate	1 (0.5)
Father’s education	Below diploma	49 (23.9)
	Diploma	54 (26.3)
	Associate	18 (8.8)
	BSc	51 (24.9)
	MSc	27 (13.2)
	Doctorate	6 (2.9)
Residence status	Dormitory	130 (63.4)
	Being with family	71 (34.6)
	Rental house	4 (2.0)
		**Mean (SD)**
Age		22.39 (2.21)
Term		6.36 (1.85)
GPA		16.65 (1.40)
Field interest		7.16 (2.04)
Study hours		8.10 (10.33)

**N**: Frequency; **%**: Percent; **HIT**: Health Information Technology; **BSc**: Bachelor of Sciences; **MSc**: Master of Sciences; **SD**: Standard Deviation; **GPA**: Grade Point Average

**Table 2:** The mean score of academic procrastination, self-esteem, moral intelligence, and their subscales of participating students

Variables		Mean	SD
Academic Procrastination	Total	81.89	13.06
	Preparing for exams	25.02	4.83
	Preparing for assignments	32.36	5.46
	Preparing for term papers	24.51	4.76
Self-Esteem	Total	5.03	4.84
	Personal competence	1.94	3.69
	Self-satisfaction	3.09	1.55
Moral Intelligence	Total	78.87	12.31
	Honesty	24.02	3.81
	Responsibility	24.59	4.45
	Forgiveness	14.49	2.97
	Sympathy	15.78	2.91

SD: Standard Deviation

**Fig. 1:**
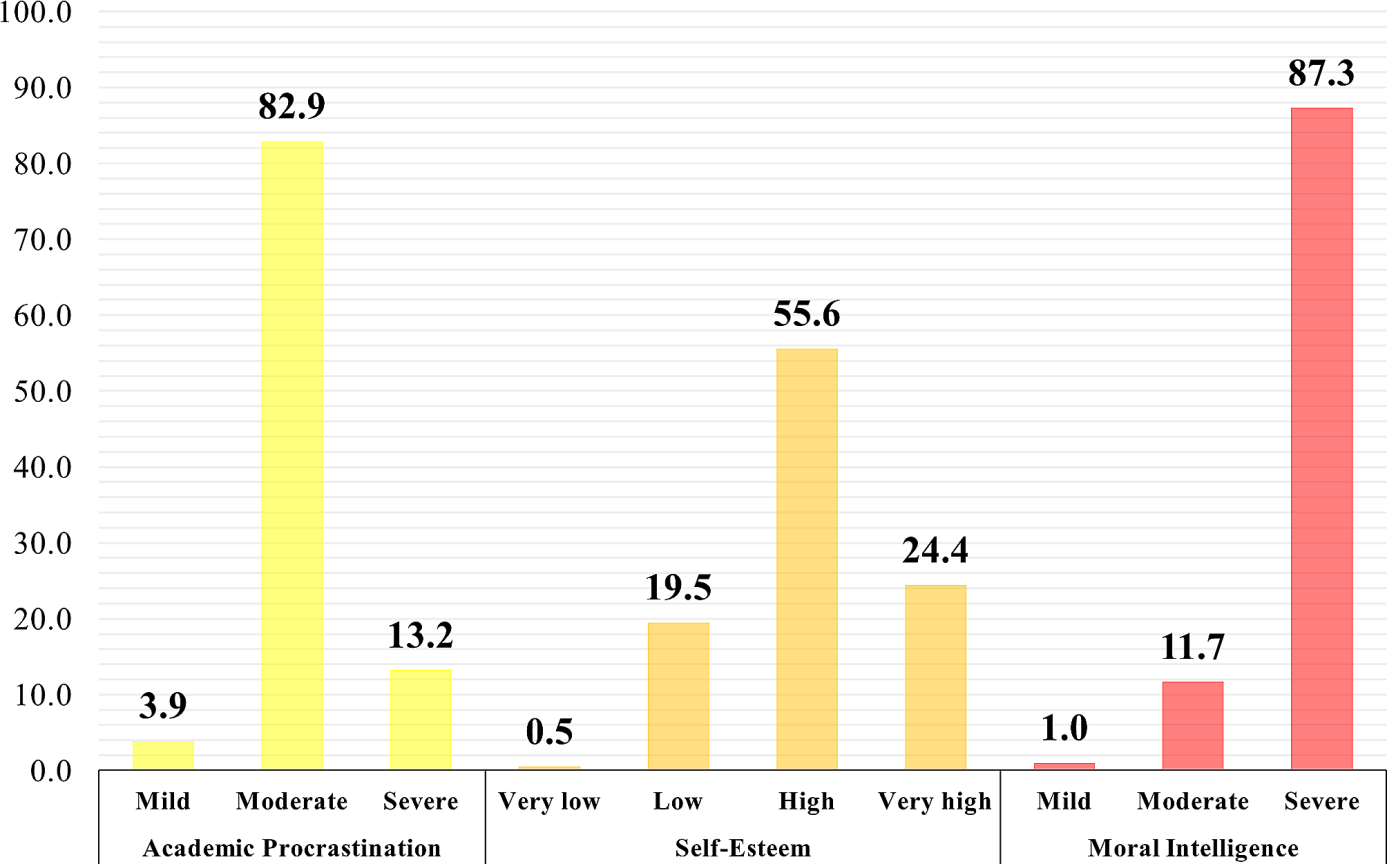
The level of academic procrastination, self-esteem, and moral intelligence of participating students

In this study, 96.1% of the students experienced moderate to severe levels of academic procrastination, and 80% of the students reported high and very high levels of self-esteem. Also, only 12.7% of the students experienced mild to moderate levels of moral intelligence. Figure 1 presents different levels of academic procrastination, self-esteem, and moral intelligence of the participating students.

The results of the backward multivariable linear regression model explained 27.7% of the variance of academic procrastination by the variables in the model. This model demonstrated that for every unit increase in self-esteem, GPA, and interest in the study field, the students’ average score of academic procrastination decreased by 0.94, 2.38, and 1.14 units, respectively (Table 3). Furthermore, no statistically significant relationship was observed between the students’ academic procrastination and moral intelligence (*P =* 0.285).

**Table 3:** The role of independent variables on academic procrastination based on a multivariate linear regression model

Model	β	SE	t	P
(Constant)	134.352	9.499	14.144	< 0.001
Self-Esteem	-0.942	0.169	-5.575	< 0.001
GPA	-2.383	0.579	-4.112	< 0.001
Field interest	-1.139	0.407	-2.800	0.006

**SE**: Standard Error; **P**: P-value; **GPA**: Grade Point Average

## Discussion

In this study, 96.1% of students experienced moderate to severe levels of academic procrastination. In a survey of students at five universities in Saudi Arabia, 7.7% and 62.8% of the participants reported severe and moderate academic procrastination, respectively [39]. Zhang et al. (2018) presented evidence that 74.1% of second to fourth-year undergraduate students of health professions were somehow involved in academic procrastination and postponed at least one of their coursework [10]. Uma et al. (2020) reported that 28.5% and 38.0% of dental students experienced severe and moderate levels of academic procrastination, respectively [29]. Accordingly, academic procrastination is among the most common problems students face. Therefore, it is necessary to highlight the importance of using appropriate strategies, such as cognitive-behavioral therapy (CBT) [40] and acceptance and commitment therapy (ACT)-based interventions [41, 42], to reduce students’ academic procrastination.

The results of this study indicated a significant negative relationship between students’ academic procrastination and self-esteem. Similarly, self-esteem is reportedly one of the factors affecting students’ academic procrastination in various studies [10, 16–18]. In this respect, lower degrees of academic procrastination were observed in students with higher self-esteem. Katz et al. (2014) claim that academic procrastination leads to adverse cognitive and emotional consequences, including a decrease in people’s self-confidence and self-esteem. As a result, those with low self-esteem procrastinate to protect themselves [43]. According to Babu et al. (2019), people with high self-esteem usually do not postpone completing assignments and tasks. In contrast, people with low self-esteem often suffer from procrastination and postpone doing tasks until the last moment [17]. In other words, students with low self-esteem often experience high levels of academic procrastination due to the fear of failure in achieving their goals. In fact, when students do not expect success and growth because of low self-esteem, they do not strive to achieve their goals [18]. Therefore, low self-esteem will result in students’ academic procrastination.

The present study showed no significant relationship between academic procrastination and students’ moral intelligence. However, Shahbaziyan et al. (2018) observed a significant negative relationship between students’ academic procrastination and moral intelligence [24]. Narimani et al. (2017) believe that students with high moral intelligence behave consistently with individual and social values and are reluctant to procrastinate. Furthermore, students with rooted honesty as a particular behavior in the depths of their souls do not postpone their work and assignments without any reasonable cause and are always regular [44]. Therefore, moral intelligence is considered a predictive and deterrent factor of procrastination and helps a person avoid procrastination and negligence [24, 44]. The obtained inconsistency may be due to the difference in the individual, cultural, and social characteristics of the studied societies and the different educational and environmental conditions at universities.

In this study, lower GPAs were recorded in students with higher levels of academic procrastination. Similar to this finding, a meta-analytical study showed a significant negative relationship between students’ academic procrastination and academic success [45]. Goroshit and Hen (2021) investigated the impact of academic procrastination on academic performance in general and specifically in students with learning disabilities (LD). The results indicated that students with LD reported lower GPAs and higher levels of academic procrastination than students without LD. In addition, a significant negative correlation was observed between students’ academic procrastination and GPA [46]. Thus, academic procrastination adversely affects students’ academic performance and many psychophysical problems and negative emotions [47].

Based on the results of this study, students with less interest in their field of study experienced higher levels of academic procrastination. According to Valizadeh et al. (2016), interest in the study field negatively affects and reduces students’ academic procrastination; in other words, less procrastination occurs in students interested in their field of study. On the other hand, when students feel more capable and efficient in doing their academic assignments and possess skill and mastery goals, they will be more interested in their field. Hence, they will not postpone tasks until the last minute [48]. Overall, it can be claimed that students who consider a task unpleasant and tedious or are not very interested in it will probably postpone doing the task. This lack of interest is also true even for students whose academic success is not affected by delaying behaviors [49].

### Research limitations and recommendations

A major limitation of this study is the use of self-reporting tools. Hence, the subjects might not have answered the questions responsibly and correctly. The large number of questions in the questionnaires and their long completion time could also have negatively affected the accuracy of the participants’ answers. Moreover, this study was conducted only on the Shahroud University of Medical Sciences students, making it difficult to generalize the results to students of non-medical sciences universities in Iran. Hence, it is suggested to conduct similar studies with a longitudinal design and a larger sample size in the future.

## Conclusion

Based on the results of this study, the majority of students experienced moderate to severe levels of academic procrastination and tended to delay their academic tasks. Furthermore, predictors of higher academic procrastination among students included low levels of self-esteem, GPA, and interest in the field of study. Therefore, improving students’ self-esteem is crucial in reducing academic procrastination. Additionally, the importance of students’ interest in their field of study in reducing issues like academic procrastination should not be overlooked. By addressing these factors, academic procrastination can be minimized, ultimately leading to improved academic outcomes such as academic performance and GPA.

## Data Availability

The datasets used and/or analyzed during the current study are available from the corresponding author upon reasonable request.

## Abbreviations

SMI: Serious Mental Illnesses
MDD: Major Depression Disorder
BD: Bipolar Disorder
OCD: Obsessive-Compulsive Disorder
PTSD: Post-Traumatic Stress
GPA: Grade Point Average
PASS: Procrastination Assessment Scale-Students
BDI: Beck Depression Inventory
ATIB: Assessment Test for Irrational Belief
RSES: Rosenberg’s Self-Esteem Scale
CFA: Confirmatory Factor Analysis
GoF: Goodness of Fit
PSE: Positive Self-Esteem
NSE: Negative Self-Esteem
DOS: Death Obsession Scale
MIQ: Moral Intelligence Questionnaire
SPSS: Statistical Package for the Social Sciences
CBT: Cognitive-Behavioral Therapy
ACT: Acceptance and Commitment Therapy
LD: Learning Disabilities
